# An eight-year national follow-up study on the employment trajectories of early-career pharmacists in Japan up to 2020

**DOI:** 10.1186/s40780-026-00602-1

**Published:** 2026-07-15

**Authors:** Takahito Ando, Takeru Shiroiwa, Shinobu Imai, Kiyohide Fushimi, Masato Yasuhara

**Affiliations:** 1 Laboratory of Pharmaceutical Care and Community Medicine, Faculty of Pharma-Sciences, Teikyo University, 2-11-1 Kaga, Itabashi-ku, Tokyo, 173-8605 Japan; 2 Center for Outcomes Research and Economic Evaluation for Health, National Institute of Public Health, Wako, Japan; 3 Division of Pharmacoepidemiology, Department of Healthcare and Regulatory Sciences, Showa Medical University School of Pharmacy, Tokyo, Japan; 4 Department of Health Policy and Informatics, Institute of Science Tokyo Graduate School of Medical and Dental Sciences, Tokyo, Japan

**Keywords:** Pharmacists, Health workforce, Longitudinal study, Career mobility, Gender differences, Japan

## Abstract

**Background:**

Geographic and sectoral maldistribution of pharmacists has become a critical challenge for Japan’s healthcare system. While the number of community pharmacists continues to rise, the chronic shortage of hospital pharmacists persists. This study aimed to clarify longitudinal career trajectories, inter-sector mobility, and predictors of leaving employment among early‑career pharmacists in Japan, using nationwide census‑based data covering all licensed pharmacists.

**Methods:**

We conducted an eight‑year longitudinal cohort study using individual‑level data from the Statistics of Physicians, Dentists and Pharmacists from 2012 to 2020. The cohort included 7,242 pharmacists (3,458 men and 3,784 women) who obtained their pharmacist license in 2011–2012 and were continuously identified in all five survey waves. Employment sectors were categorized into 10 groups. Using generalized estimating equations (GEE), we analyzed the odds of being “unemployed” (defined as having no institutional affiliation), adjusting for gender, survey year, and age.

**Results:**

At baseline (2012), hospital and community pharmacists accounted for 84.6% of the cohort. By 2020, the proportion of community pharmacists had increased, whereas the proportion of hospital pharmacists had substantially declined. Among those initially employed in hospitals, 34.5% left the hospital sector within eight years, and 83.6% of these individuals transitioned to community pharmacies. In contrast, 88.9% of community pharmacists remained in the same sector throughout the study period. The proportion unemployed among men remained below 1.0%, while the rate among women increased from 0.4% to 3.7%. GEE analysis demonstrated that women had a significantly higher ratio of being unemployed over time compared with men, independent of age (adjusted odds ratio 1.203, 95% CI: 1.118–1.293, *p* < 0.001).

**Conclusions:**

A substantial outflow from hospitals to community pharmacies was observed among early‑career pharmacists in Japan, suggesting that the hospital pharmacist shortage results not only from recruitment challenges but also from significant retention issues during the career continuation process. Additionally, women exhibited a distinctively higher ratio of career interruption. Policies that enhance retention through incentives for specialization in hospitals, along with targeted support for women’s career continuity, are essential for sustaining the pharmacist workforce.

**Supplementary information:**

The online version contains supplementary material available at https://doi.org/10.1186/s40780-026-00602-1.

## Background

The geographical maldistribution of pharmacists is a critical global healthcare challenge. The International Pharmaceutical Federation (FIP) has pointed out that the excessive concentration of pharmacists in urban areas and pharmacist shortages in rural regions are widening disparities in healthcare access [1]. This maldistribution is one of the factors that weakens regional healthcare systems, and research into the elucidation of these factors and corrective measures is being conducted in various countries [2–5].

One of the important factors defining the regional maldistribution of pharmacists is turnover and attrition (inter-sectoral mobility). The World Health Organization (WHO) emphasizes the importance of measures against the turnover of healthcare workers; while high turnover rates lead to reduced organizational productivity and loss of knowledge, they also have aspects such as better matching individual skills with workplace needs [6]. According to the FIP, job satisfaction is a primary indicator of turnover and retention rates [1], and analyses of the turnover intentions of pharmacists in various countries have been reported [7].

In Japan, the maldistribution of pharmacists is a serious issue. According to the “Pharmacist Distribution Index” published by the Ministry of Health, Labour and Welfare (MHLW), while there is a nationwide shortage of hospital pharmacists, the situation for community pharmacists is one where sufficient and insufficient areas coexist [8]. This maldistribution is characterized by both geographical disparities, such as shortages in rural areas, and inter-sectoral imbalances, particularly the insufficient number of hospital pharmacists. Such inter-sectoral imbalances are a common challenge internationally; for instance, the proportion of community pharmacists and hospital pharmacists differs greatly between Europe and Southeast Asia [1]. To achieve appropriate pharmacist procurement and sustainable placement, it is necessary to scientifically verify whether the factors behind the pharmacist shortage stem from a lack of absolute numbers or from human resource outflow.

In Japan, the “Statistics of Physicians, Dentists and Pharmacists,” covering all pharmacists, is conducted biennially, and all pharmacists are legally mandated to report their current residence, workplace, and employment sector. Each pharmacist is assigned a “Pharmacist Registration Number”—a lifelong, immutable identifier—upon licensure. Using this number as a key, it is possible to track specific individuals over a long period. In this study, using individual-level data from the same statistics, we extracted a cohort of early-career pharmacists who had been registered for less than two years as of 2012 (new registrants in 2011 and 2012) and conducted an 8-year longitudinal study through 2020. This study aimed to clarify the career trends, inter-sectoral mobility, and factors associated with the departure from the workforce among early-career pharmacists in Japan.

## Methods

### Data source and database construction

**Fig. 1:**
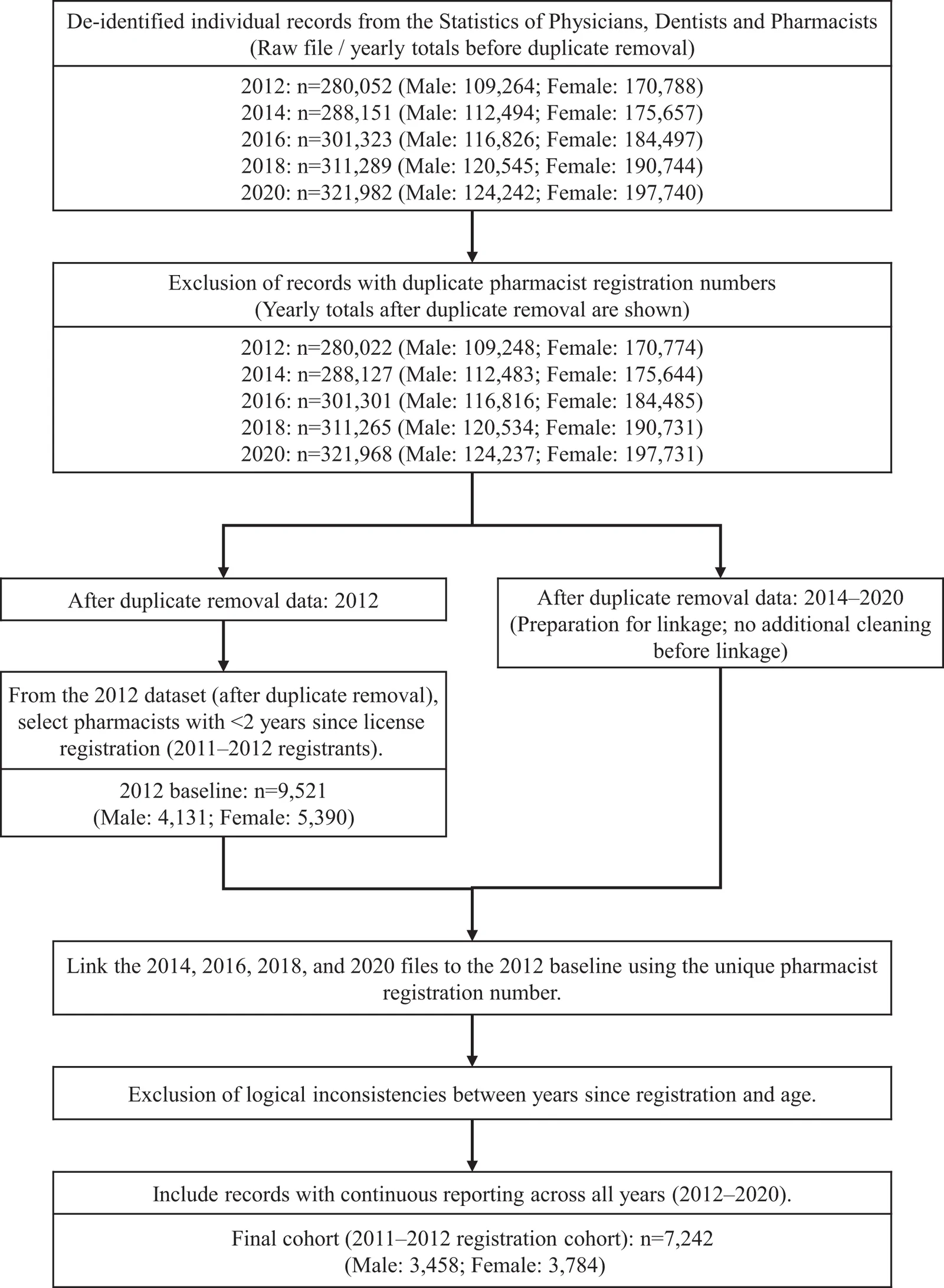
Flowchart of data cleaning and construction of the 2011–2012 registration cohort. This flowchart illustrates the data cleaning process and the establishment of the baseline cohort. Individual-level records from the Statistics of Physicians, Dentists, and pharmacists (2012–2020) were first deduplicated; yearly totals before and after deduplication are presented. From the cleaned 2012 dataset, pharmacists with less than two years since license registration (2011–2012 registrants) were selected to form the baseline (*n* = 9,521). The 2014, 2016, 2018, and 2020 datasets were then linked to this baseline using unique registration numbers without further selection. After excluding records with logical inconsistencies between age and years since registration, the final cohort comprised 7,242 pharmacists (3,458 males and 3,784 females) with continuous reporting across all survey years (2012–2020)

Individual-level data from the “Statistics of Physicians, Dentists and Pharmacists” (2012, 2014, 2016, 2018, and 2020) provided by the Ministry of Health, Labour, and Welfare were used. This survey was a census covering all pharmacists in Japan. The extraction algorithm for the analysis targets is illustrated in Fig. 1. First, duplicate records for the same pharmacist registration number were removed from the data for each year. Next, the early-career pharmacists who registered in 2011 or 2012 were selected from baseline 2012 survey and linked to the 2014, 2016, 2018, and 2020 data using the pharmacist registration number as the key. After linkage, data inconsistent in terms of years since registration and age (data that increased or decreased significantly over time) were excluded. Finally, 7,242 individuals (3,458 males, 3,784 females) whose reports were continuously confirmed across all five surveys until 2020 were extracted as the “target pharmacists.”

### Reclassification of employment sectors

To ensure longitudinal comparability, the sub-classifications of employment sectors, which differed across survey years, were integrated and unified into the following 10 categories based on the study-specific criteria of this study:

1) Community pharmacists (owners and employees), 2) Hospital pharmacists (including clinics), 3) University, 4) Graduate students, etc., 5) Pharmaceutical manufacturing industry, 6) Pharmaceutical sales industry, 7) Health administration, 8) Others (including those working in nursing care facilities), 9) Unemployed, and 10) Unknown. Note that “nursing care facilities,” which was added from 2018 onwards, was included in “Others” to maintain consistency. Regarding employment status, although a distinction between full-time and part-time employment was introduced in 2016, this study did not analyze these patterns to maintain longitudinal consistency from the 2012 baseline. Consequently, all pharmacists affiliated with a facility were categorized by sector, regardless of their specific employment contract or hours.

### Statistical analysis

Starting from the employment sector as of 2012 (Pharmacy or Hospital), we longitudinally observed inter-sectoral mobility and transitions to an unemployed state over the 8-year period until 2020. To verify gender differences in the transition to an unemployed state, an analysis using Generalized Estimating Equations (GEE) was conducted, with employment status at each survey point (unemployed = 1, employed = 0) as the dependent variable. The independent variables included gender (male = 0, female = 1), survey year (time), and age. Furthermore, to evaluate changes in gender differences over time, an interaction term between gender and survey year (gender × time) was included in the model. To control for correlations due to repeated measurements within individuals, a first-order autoregressive (AR(1)) structure was selected for the working correlation structure, and a logit function was adopted as the linking function. IBM SPSS Statistics Version 29 was used for statistical analysis, and the significance level was set at less than 5% (two-sided).

## Results

### Attributes of the early-career pharmacist cohort and 8-year trends

**Fig. 2:**
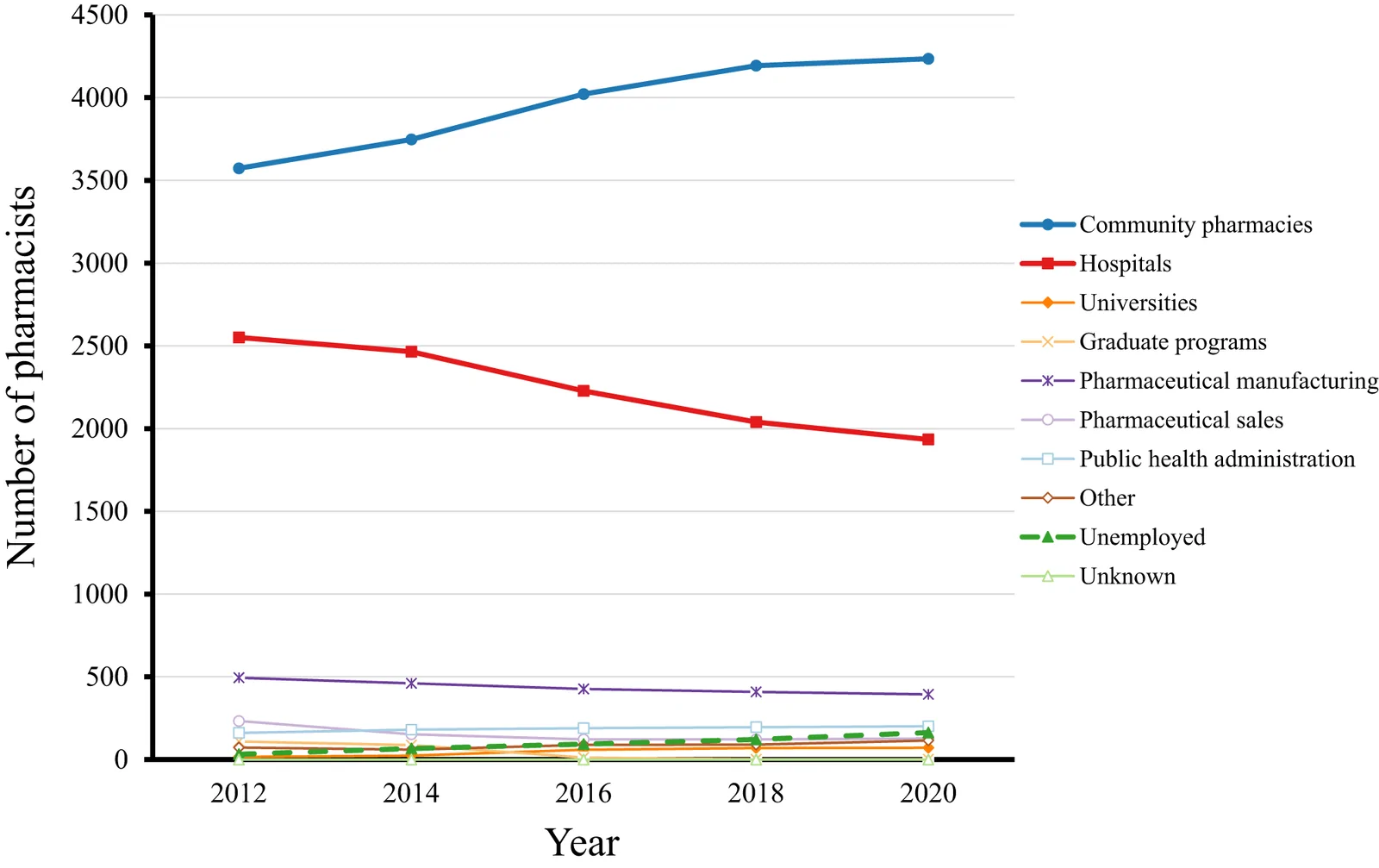
Changes in the number of pharmacists by employment sector (2012–2020). This figure illustrates the biennial changes in the number of pharmacists across ten employment sectors. A continuous increase was observed in the community pharmacy sector, whereas the number of hospital pharmacists exhibited a declining trend. Additionally, the number of unemployed pharmacists increased over the 8-year period. Detailed numerical data for all sectors are available in Supplementary Table S1

**Fig. 3:**
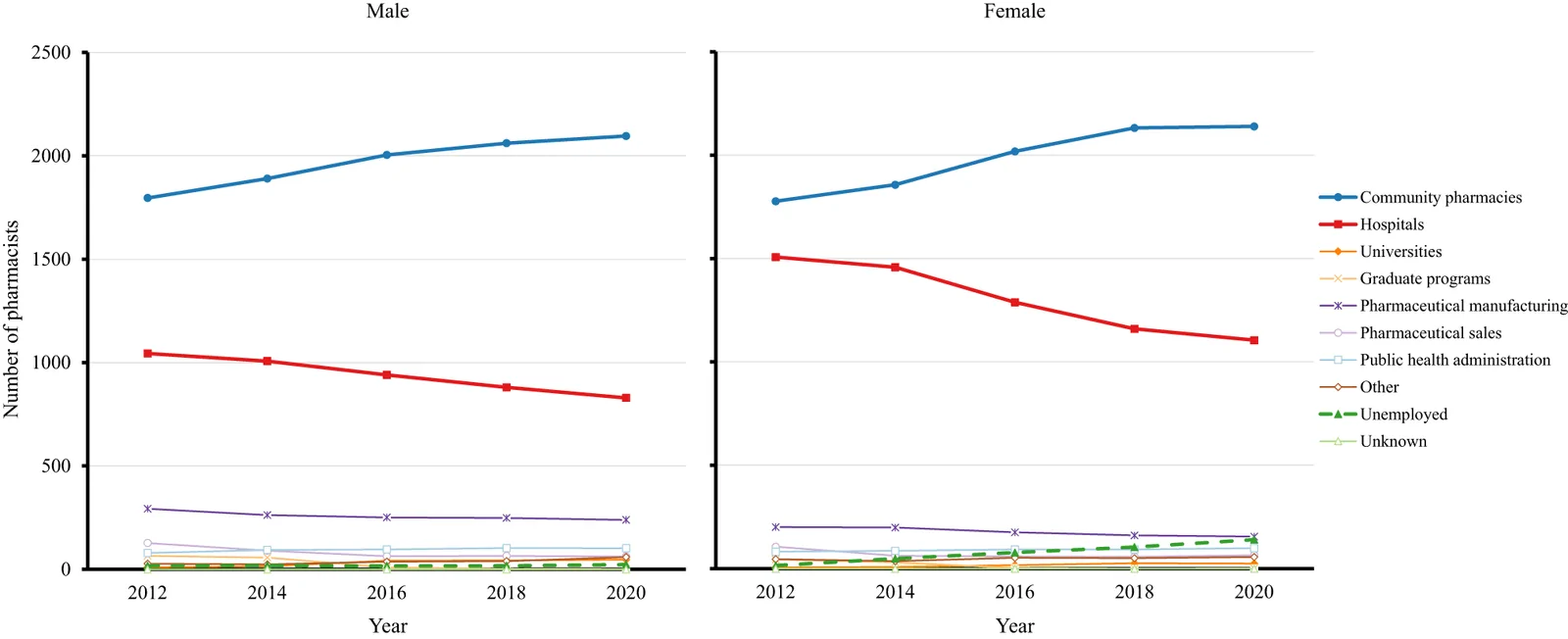
Changes in the number of pharmacists by employment sector, stratified by gender (2012–2020). This figure illustrates the biennial changes in pharmacist numbers across ten employment sectors for men (left) and women (right). In both genders, an increase was observed among community pharmacists, while hospital pharmacists exhibited a declining trend. Notably, among women, the number of unemployed pharmacists also showed a continuous upward trend over the 8-year period. Detailed numerical data for all sectors are available in Supplementary Table S2

As shown in Figs. 2 and 3, notable shifts in employment sectors and gender-specific differences were observed over the eight-year period. The breakdown by sector of the 7,242 targets as of 2012 was as follows: 3,573 community pharmacists (1,796 males, 1,777 females), 2,551 hospital pharmacists (1,044 males, 1,507 females), 16 university employees (10 males, 6 females), 109 graduate students, etc. (65 males, 44 females), 494 in pharmaceutical manufacturing (293 males, 201 females), 233 in pharmaceutical sales (127 males, 106 females), 161 in health administration (79 males, 82 females), 73 others (27 males, 46 females), 32 unemployed (17 males, 15 females), and 0 unknown. The two sectors of hospital and community pharmacists accounted for approximately 85% of the total. Looking at the employment sectors in 2020, eight years later, community pharmacists increased to 4,235 (2,096 males, 2,139 females). Conversely, hospital pharmacists had decreased to 1,934 (829 males, 1,105 females). Additionally, the number of unemployed individuals increased to 163 (23 males, 140 females), with the increase being particularly prominent among women.

Notably, gender differences were observed in the transition of the unemployment rate. The unemployment rate for males remained at approximately 0.5% and was almost constant from 2012 (0.5%) to 2020 (0.7%). In contrast, the unemployment rate for females showed an increasing trend over time: 0.4% in 2012, 1.3% in 2014, 2.1% in 2016, 2.7% in 2018, and 3.7% in 2020.

### Career trends of community pharmacists

**Fig. 4:**
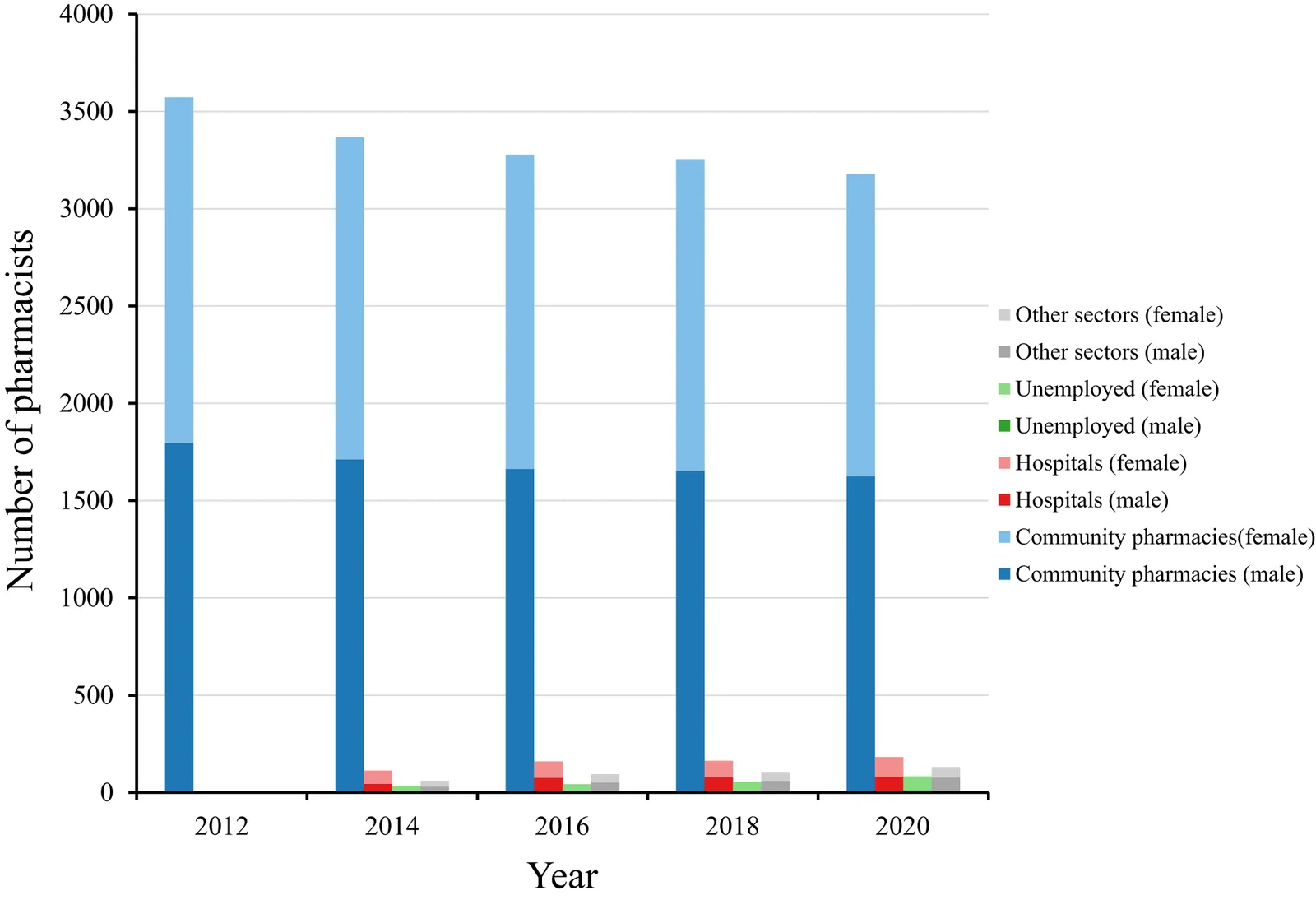
Changes in employment sector (2012–2020) among pharmacists working in community pharmacies in 2012. This figure tracks the biennial transitions in employment sectors from 2012 to 2020 for the cohort of pharmacists employed in community pharmacies in 2012. While the number of those remaining in community pharmacies declined over time, increases were observed in other sectors and among unemployed pharmacists. The category “Other sectors” comprises universities, graduate programs, pharmaceutical manufacturing, pharmaceutical sales, public health administration, other, and unknown. Detailed numerical data are available in Supplementary Table S3

As a result of tracking the 3,573 individuals who were employed in pharmacies in 2012 until 2020, 3,177 (88.9%) continued to work in pharmacies, showing a high continuation rate. Among the 396 individuals who moved to other sectors, the most common destination was the hospital (182 individuals) (Fig. 4).

### Career trends of hospital pharmacists

**Fig. 5:**
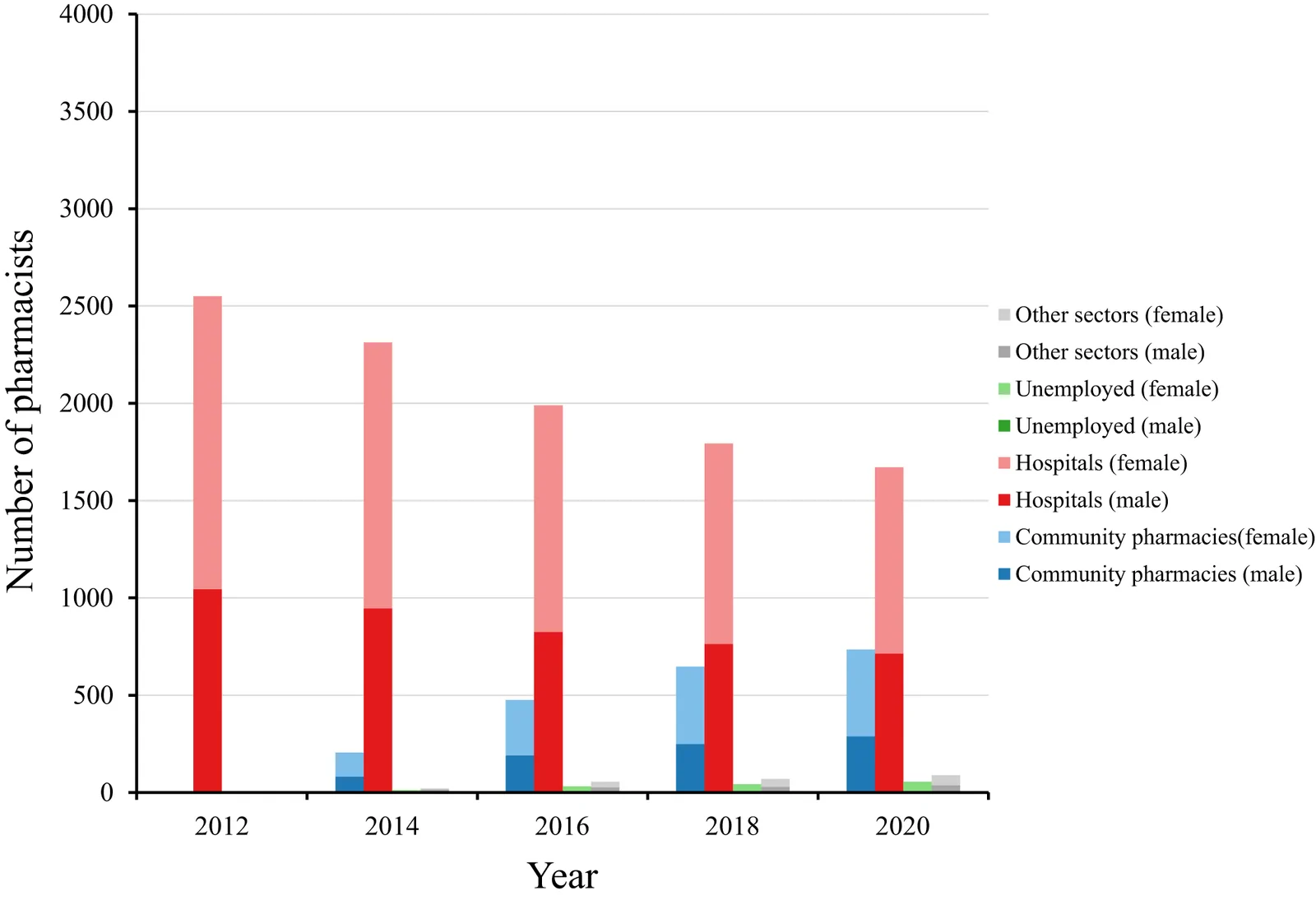
Changes in employment sector (2012–2020) among pharmacists working in hospitals in 2012. This figure tracks the biennial transitions in employment sectors from 2012 to 2020 for the cohort of pharmacists employed in hospitals in 2012. While the number of those remaining in hospitals decreased over time, an increase was observed in community pharmacies, other sectors, and among unemployed pharmacists. The category “Other sectors” comprises universities, graduate programs, pharmaceutical manufacturing, pharmaceutical sales, public health administration, other, and unknown. Detailed numerical data are available in Supplementary Table S4

Of the 2,551 individuals who were employed in hospitals in 2012, only 1,672 (65.5%) remained in hospital employment as of 2020. Among the 879 who moved to other sectors, the most common destination was community pharmacies (735 individuals), accounting for more than 80% (83.6%) of those who moved. A large-scale mobility of human resources from hospitals to pharmacies was confirmed in the early career stage (Fig. 5).

### Generalized estimating equations (GEE) analysis of unemployment status

The GEE analysis with unemployment status as the dependent variable showed a statistically significant difference in the interaction term between gender (Gender) and survey year (Time) (*p* < 0.001). This indicates that even after adjusting for the effects of age, the rate of increase in the odds of being unemployed over time is significantly greater for females than for males. Specifically, the adjusted odds ratio (aOR) for the interaction term was 1.203 (95% CI: 1.118–1.293), indicating that the odds of being unemployed among female pharmacists increased by a factor of 1.203 every two years relative to their male counterparts. The analysis results quantitatively showed that in the Japanese early-career pharmacist cohort, females have a significantly higher ratio of leaving the workforce as their careers progress compared to males, independent of age factors (Table 1).

**Table 1:** Longitudinal analysis of factors associated with unemployment among pharmacists (GEE model)

Variable	B (Log Odds)	Adjusted Odds Ratio (aOR)	95% Confidence Interval (CI)	*p*-value
Main Effects				
Time (per 2-year increase)	−0.043	0.958	[0.892, 1.028]	0.236
Gender (Female vs. Male)	−371.601	<0.001	[<0.001, <0.001]	<0.001***
Age (at each time point)	0.078	1.081	[1.045, 1.117]	<0.001***
Interaction Effect				
Time×Gender (Female)	0.185	1.203	[1.118, 1.293]	<0.001***

Abbreviations: GEE, generalized estimating equations; aOR, adjusted odds ratio; CI, confidence interval.Notes: The analysis used a logit link function and an AR(1) working correlation structure. aOR was calculated as exp(*B*). Male (Gender = 0) served as the reference category.*** *p* < 0.001

The GEE analysis revealed a significant interaction between time and gender (adjusted odds ratio [aOR] = 1.203; 95% confidence interval [CI]: 1.118–1.293; *p* < 0.001). This indicates that among female pharmacists, the odds of being unemployed increased by a factor of 1.203 every two years relative to their male counterparts, after adjusting for age

## Discussion

This study tracked a cohort of early-career pharmacists, including the first generation to receive 6-year education, over eight years and clarified the labor market trends of pharmacists in Japan based on census data. The results of this study highlighted that while a substantial proportion of young pharmacists choose hospital employment in their early careers, a large-scale outflow of human resources from hospitals to community pharmacies occurs over time, and the ratio of leaving the workforce (becoming unemployed) is uniquely high among female pharmacists.

### Flux from hospital to community pharmacists and acceleration of maldistribution

The cohort in this study, consisting of the group with less than two years of experience as of 2012, includes many from the first graduating class (class of 2012) of the 6-year pharmaceutical education program that began in 2006 [9]. This generation serves as a benchmark group for the sector selection of pharmacists to be trained in the future. The analysis showed that while the proportion of hospital pharmacists was relatively high in the early career compared to later years, the gap between hospital and pharmacy employment widened over time as the number of individuals moving from hospitals to pharmacies significantly exceeded those moving from pharmacies to hospitals.

**Fig. 6:**
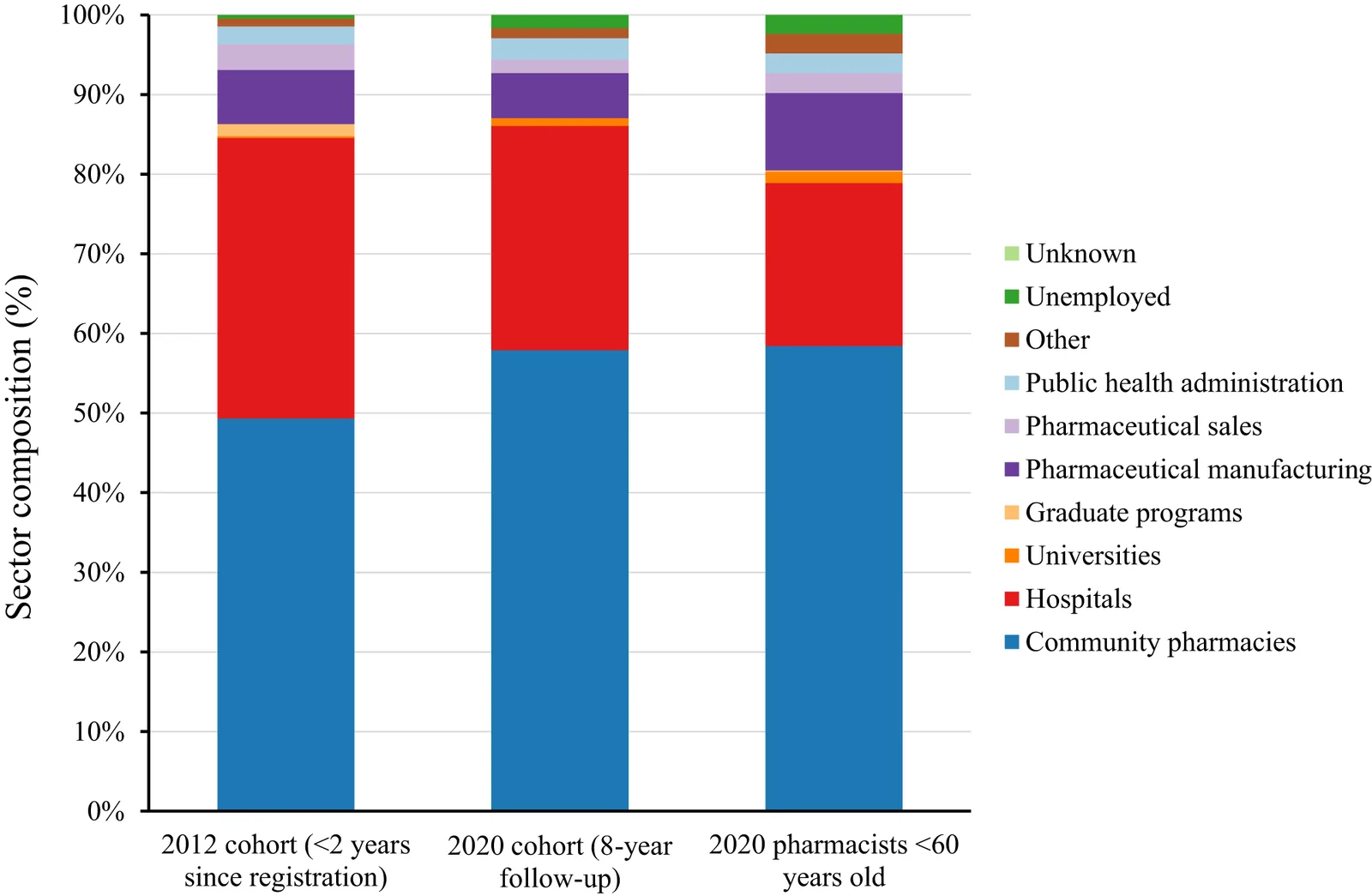
Sector composition in the 2012 and 2020 cohorts and pharmacists under age 60 in 2020. This figure compares the employment sector composition among three pharmacist groups: (1) the 2012 baseline cohort (pharmacists with less than two years since license registration), (2) the same cohort in 2020 after eight years of follow-up, and (3) pharmacists under age 60 in 2020. Each bar represents 100% of the respective group and illustrates the proportions of major employment sectors. The group aged < 60 years was constructed as a reference population by extracting pharmacists directly from the deduplicated 2020 dataset of the Statistics of Physicians, Dentists, and pharmacists, following the initial cleaning process outlined in Fig. 1

Interestingly, the proportion of hospital employees among early-career pharmacists was higher than the overall statistical values for all pharmacists. However, this proportion decreased year by year, showing a trend toward converging with the average compositional ratio of all pharmacists (Fig. 6). This suggests that the current serious shortage of hospital pharmacists in Japan is maintained and strengthened not only by a shortage at the time of entry but also by outflow during the process of career continuation. Therefore, to resolve the hospital pharmacist shortage, it is indispensable to address both the strengthening of new recruitment and the suppression of outflow (retention) among early-career and mid-career pharmacists. Japan’s population structure is facing rapid aging and a declining birthrate, with the aging rate predicted to reach 37.9% by 2060 [10]. This demographic change brings about qualitative and quantitative transformations in healthcare demand due to the complication of disease structures, making it difficult to maintain the healthcare provision system, especially in rural areas. The continuous outflow of human resources from hospitals shown in this study suggests that the supply system for hospital pharmacists may collapse in the face of increasing future healthcare needs.

A major factor promoting such human resource outflow is the disparity in treatment between hospitals and pharmacies. Previous studies have shown that economic factors such as salary and treatment are directly linked to pharmacists’ job satisfaction and turnover risk [11–14], and the gap in expected salary, including the starting salary, has a decisive impact on the sector selection of early-career pharmacists [15]. In Japan’s medical fee system, there is a structural challenge where the professional contribution of pharmacists is difficult to visualize as hospital revenue [16], and the current situation where hospital salary levels are low relative to the weight of responsibility is pushing the flux. In recent years, Japan’s medical fee system has begun to evaluate the placement of pharmacists with specialized qualifications [17], but it is essential to have a perspective that specifically links such systemic evaluations to hospitals’ own salary structures and promotion opportunities. Therefore, hospital management should redefine pharmacists as “strategic human resources for ensuring appropriate medical quality” and improve retention rates by strengthening economic and organizational incentives for pharmacists with advanced professional skills.

### Career interruptions of female pharmacists and utilization of potential labor force

The GEE analysis in this study quantitatively showed that female pharmacists had a significantly higher ratio of leaving the workforce (becoming unemployed) than males. While the unemployment rate for males was almost constant over the eight years, it increased over time for females, highlighting the significant impact of life events on female career continuation.

At the start of this study’s cohort in 2012, the “M-shaped curve,” where the female labor force participation rate in Japan drops during the marriage and child-bearing period, was prominent, and leaving the workforce due to life events was a common social background [18]. According to recent reports, the dip in the M-shaped curve is being resolved, and the continuous employment rate for women is on an upward trend. However, it remains lower than the employment rate for men, and there remains the issue of the “L-shaped curve,” where the regular employment rate peaks at ages 25–29 and then declines as individuals transition to non-regular employment [19]. Despite the government’s promotion of women’s social advancement, our GEE analysis results confirm that these structural career interruption trends among female pharmacists persisted throughout the survey period from 2012 to 2020. In the case of pharmacists, it remains unclear whether this L-shaped curve is primarily attributed to complete career interruption or a choice of more flexible employment patterns; this cannot be definitively determined without further granular analysis. While the present study did not evaluate these employment types due to the lack of longitudinal data consistency since the 2012 baseline, investigating the transition between full-time and part-time status remains a crucial task for future research.

According to previous research in the United States, female pharmacists have a higher annual turnover rate than males, with a high proportion citing “relocation” as the main reason, whereas males tend to emphasize “salary” and a “desire for change” [20]. Furthermore, the “paradox of the contented female worker,” where women have higher career satisfaction even with lower income than men, has also been pointed out [21]. Based on this finding, while the satisfaction of male pharmacists is influenced by monetary rewards such as annual income, female pharmacists are likely to select their fields of work by emphasizing non-monetary factors such as balance with private life, proximity to home, and acquisition of clinical skills [21].

Such gender differences in career selection are also closely related to the “geographical maldistribution” in Japan. Our previous research clarified that early-career female pharmacists tend to concentrate in urban areas more than males [22]. This is inferred to be a result of women aiming for urban areas where employment options are abundant and flexible working styles are possible, anticipating the transition to non-regular employment symbolized by the L-shaped curve and interruptions/re-employment due to life events. The combination of the high ratio of leaving the workforce shown in this study and the urban concentration trend is further exacerbating the pharmacist shortage in rural areas and hospitals.

The career interruption of female pharmacists is not merely an individual problem but a loss of the primary labor force that accounts for more than half of all pharmacists in the country. Similar barriers have been reported for female physicians and nurses [23, 24], indicating a structural problem across healthcare professions. Therefore, to utilize potential pharmacists as a valuable labor force, in addition to improving monetary treatment, policy interventions that align with women’s professional values and actual situations—such as support for maintaining and improving clinical skills, development of flexible working forms tailored to life bases, and support for returning to work as regular employees—are indispensable.

### Pharmacist supply and demand forecast and future outlook

In the supply and demand forecast by the Ministry of Health, Labour and Welfare, an “oversupply” of pharmacists is predicted in the future [25]. However, as shown by the results of this study, the maldistribution between sectors and regions has not yet been resolved [8]. Even if the total absolute number becomes sufficient, there is a high possibility that “qualitative and local shortages” in specific sectors, such as hospitals and rural areas, will not be resolved.

In the future, as the labor market transitions from a “seller’s market” to a “buyer’s market,” hospitals must present a clear professional career path for pharmacists, not just as a place of work. Aligning individual pharmacists’ career development with hospital management incentives will be the key to sustainable human resource procurement. For example, overseas studies have demonstrated that clinical pharmacists’ interventions not only improve patient safety but also generate significant economic benefits for hospital management through cost avoidance and direct cost savings [26, 27]. Furthermore, integrating staff pharmacists into clinical practice models [28] and deploying them in specialized settings such as operating rooms [29] have been shown to reduce medical supply costs and improve overall surgical efficiency, thereby aligning professional advancement with institutional financial goals.

### Policy implications

Based on the above findings, we propose the following two comprehensive approaches to correct pharmacist maldistribution and future labor shortages in Japan.

First, an appropriate evaluation system for hospital pharmacists’ professionalism should be established, along with strengthening economic support based on it. A mechanism should be established where pharmacists’ career advancement (acquisition of specialized/certified pharmacist status, etc.) contributes directly to hospital management through medical fees, and this model should be directly linked to the improvement of individual pharmacists’ treatment. This will serve as a strong motivation (retention strategy) for pharmacists to continue choosing hospital employment as an attractive career path in the future, where an oversupply is predicted.

Second, return-to-work and retention support that views female pharmacists’ career continuation and geographical maldistribution as a set. In view of the current situation where female pharmacists concentrate in urban areas and have a high ratio of leaving the workforce, the introduction of flexible working forms in regional hospitals and the development of smooth return-to-work support programs after life events are required. In particular, in the super-aging society toward 2060, policy intervention to relocate inactive pharmacists as “high-quality medical resources” to rural areas and hospitals will be the key to increasing the sustainability of the healthcare provision system.

## Limitations

There are several limitations to consider when interpreting the results of this study.

First, this study is a secondary analysis based on the report data of the “Statistics of Physicians, Dentists and Pharmacists,” and it cannot grasp the “direct motivations” of individual pharmacists who chose to leave the workforce or move between sectors. The influence of economic incentives and life events (marriage, childbirth, childcare, etc.) by gender discussed in the discussion are theoretical inferences based on previous research and Japan’s socio-economic situation, and they do not directly link individual motivations with behaviors.

Second, this study did not categorize community pharmacies based on their locational or functional characteristics. In Japan, as discussed in the contexts of pharmaceutical fee revisions by the Central Social Insurance Medical Council (Chuikyo), pharmacies are often distinguished by their settings, such as those located near hospitals (“monzen” pharmacies) or those serving as community-based pharmacies. Since working hours and professional roles can vary depending on these environments, such factors could potentially influence career continuity and inter-sectoral mobility. However, because the “Statistics of Physicians, Dentists and Pharmacists” classifies all pharmacies as a single category, it was not possible to analyze the impact of these locational differences. Future studies utilizing more granular data on pharmacy settings are needed to provide a more nuanced understanding of pharmacist labor dynamics.

Third, the distribution of employment sectors at the 2012 baseline may have been influenced by the specific labor market conditions of that period. The transition to the six-year pharmacy education program resulted in an exceptionally low number of new graduates in 2010 and 2011, which may have led to a surge in hospital job openings around 2012. It is possible that this unique recruitment environment, immediately following a period of supply shortage, influenced the subsequent career trajectories and sectoral shifts observed in this study.

Fourth, there is a potential for misclassification regarding pharmacists on temporary leave, such as maternity or childcare leave. While such leave can be considered a form of “leaving the workforce,” specific survey items regarding leave were only introduced in 2016. Our careful verification of the 2016–2020 dataset confirmed that individuals on leave were categorized under their respective employment sectors rather than as “unemployed.” However, as this classification cannot be definitively guaranteed for the 2012 and 2014 surveys, a degree of ambiguity remains for the first two survey cycles. Consequently, while the “unemployed” status in later years reflects a genuine career interruption, the data for 2012 and 2014 should be interpreted with caution regarding the potential inclusion of temporary leave recipients.

Fifth, the analysis targets of this study were limited to pharmacists whose reports could be continuously confirmed in all five surveys from 2012 to 2020, and those who became untraceable were excluded. Under the Japanese system, holders of a pharmacist license are mandated to report every two years; however, there is a possibility that pharmacists not engaged in practice (unemployed) did not comply with this mandate. Additionally, reporting may be difficult for those living overseas, and the specific reasons for these untraceable groups cannot be identified from these data. Therefore, there is a possibility that those who left the workforce or moved overseas are included among the untraceable cases, and the results for the proportion of unemployed persons and career trends in this study may include a selection bias.

In the future, to complement the quantitative trends obtained in this study, qualitative verification through questionnaire and interview surveys targeting early-career and mid-career pharmacists is necessary.

## Conclusion

Through tracking census data over an 8-year period from 2012 to 2020, this study quantitatively clarifies for the first time the actual state of large-scale inter-sectoral mobility among early-career pharmacists in Japan and the significant gender differences in leaving the workforce (becoming unemployed).

The analysis results showed that approximately 35% of early-career pharmacists who chose hospital employment in 2012 left or moved within eight years, with the majority flowing into pharmacy. This finding suggests that the current shortage of hospital pharmacists is amplified not only by a shortage of new entrants but also by a structural outflow during the career development process. Furthermore, the GEE analysis confirmed that female pharmacists face a significantly higher ratio of leaving the workforce than male pharmacists, independent of age factors. Combined with the findings of previous research on the urban concentration of female pharmacists, this high risk of leaving the workforce is likely to be a factor further exacerbating the maldistribution of pharmacists in rural areas and hospitals.

## Electronic supplementary material

Below is the link to the electronic supplementary material.

Supplementary Material 1

Supplementary Material 2

Supplementary Material 3

Supplementary Material 4

## Data Availability

The data supporting the findings of this study were provided by the Ministry of Health, Labour and Welfare (MHLW) of Japan. These data were used under a license for the current study and were not publicly available. However, for researchers with legitimate rationale, the authors can provide guidance on the application procedures required to request data access from the MHLW.
